# Leveraging emergency department visits to connect older adults at risk for malnutrition and food insecurity to community resources: design and protocol development for the BRIDGE study

**DOI:** 10.1186/s40814-020-00576-3

**Published:** 2020-03-03

**Authors:** Andrea M. Morris, Jessa K. Engelberg Anderson, Brenda Schmitthenner, Aileen F. Aylward, Rayad B. Shams, Karen Hurka-Richardson, Timothy F. Platts-Mills

**Affiliations:** 1 West Health Institute, La Jolla, CA USA; 2 Department of Emergency Medicine, University of North Carolina, 170 Manning Drive CB #7594, Chapel Hill, NC 27599 USA

**Keywords:** Geriatrics, Emergency medicine, Malnutrition, Food assistance, Home care services, Social determinants of health

## Abstract

**Background:**

Malnutrition is a complex and costly condition that is common among older adults in the United States (US), with up to half at risk for malnutrition. Malnutrition is associated with several non-medical (i.e., social) factors, including food insecurity. Being at risk for both malnutrition and food insecurity likely identifies a subset of older adults with complex care needs and a high burden of social vulnerability (e.g., difficulty accessing or preparing meals, lack of transportation, and social isolation). US emergency departments (EDs) are a unique and important setting for identifying older patients who may benefit from the provision of health-related social services. This paper describes the protocol development for the Building Resilience and InDependence for Geriatric Patients in the Emergency Department (BRIDGE) study. BRIDGE was designed to assess the feasibility of an ED-based screening process to systematically identify older patients who are at risk for malnutrition and food insecurity and link them to health-related social services to address unmet social needs and support their health and well-being.

**Methods:**

Phase 1 efforts will be formative and focused on identifying screening tools, establishing screening and referral workflows, and conducting initial feasibility testing with a cohort of older patients and ED staff. In phase 2, which includes process and outcome evaluation, the screening and referral process will be piloted in the ED. A partnership will be formed with an Area Agency on Aging (AAA) identified in phase 1, to assess resource needs and identify community-based social services for older ED patients who screen positive for both malnutrition risk and food insecurity. Data on screening, referrals, linkage to community-based social services, and patient-reported quality of life and healthcare utilization will be used to assess feasibility.

**Discussion:**

The tools and workflows developed and tested in this study, as well as learnings related to forming and maintaining cross-sector partnerships, may serve as a model for future efforts to utilize EDs as a setting for bridging the gap between healthcare and social services for vulnerable patients.

## Background

Malnutrition is common among older adults [1] and contributes to poor health and premature death [2–5]. The annual economic burden of disease-associated malnutrition in the United States (US) is over $155 billion, with more than $51 billion of that cost attributable to older adults [6, 7]. Malnutrition is a complex, multifaceted condition and is associated with numerous risk factors that are often synergistic or bidirectional in nature. Clinical risk factors include, but are not limited to, polypharmacy, chronic medical conditions, depression, poor oral health, impaired swallowing, and frailty. Non-medical (i.e., social) risk factors include, but are not limited to, lack of transportation, loneliness and social isolation, low-quality diets, and food insecurity [6, 8, 9].

Food insecurity (i.e., lack of access or inability to afford adequate food) is a critical modifiable social risk factor for malnutrition and is a common problem among older adults in the US [10]. Food-insecure older adults have high rates of medical problems such as diabetes, depression, and heart disease, and food insecurity likely contributes to worse health outcomes [11]. Older adults with complex medical needs often also have social needs that can contribute to increased risk for both malnutrition and food insecurity. Breaking the cycle of food insecurity leading to worse health outcomes resulting in worsening food insecurity is essential to optimize health outcomes for these patients. One approach to addressing social needs is through existing community-based social services, which can be leveraged to help prevent or reduce risk in community-dwelling older adults. For example, though it is underutilized [12], there is evidence that enrolling older adults in food assistance programs, such as the Supplemental Nutrition Assistance Program (SNAP), can improve health status and enable patients to remain in their homes [7, 13]. There is also evidence that home-delivered meal programs, which serve a largely homebound older adult population, can improve nutritional status while also reducing social isolation and falls [7, 14]. For particularly vulnerable populations, such as those transitioning from hospital to home, medically tailored home-delivered meals are associated with decreased healthcare utilization and reduced spending on medical care [15, 16].

Using a clinical setting, such as an emergency department (ED), to screen for malnutrition risk and food insecurity may be an effective “foot in the door” approach that has the potential to improve health outcomes and decrease healthcare utilization [7, 13, 17]. EDs in the US receive over 20 million visits by older adults annually and are a common and essential site of care for individuals with limited financial resources [18]. Recent estimates indicate that approximately 15% of older patients seeking ED care are malnourished, and almost half are either malnourished or at risk of malnutrition [1, 6]. Further, food insecurity was identified as an important contributing factor to malnutrition for more than 25% of ED patients with malnutrition [1, 6]. Thus, based on these estimates, a national ED-based screening intervention has the potential to impact over 500,000 affected older adults.

Despite the potential impact of screening for malnutrition and food insecurity, implementing a new process in an ED setting, where time is at a premium, may be challenging and require special considerations to avoid overburdening the already busy staff. In addition, while the existing staff is clinically trained, they may lack knowledge of social risk factors for malnutrition, including food insecurity, and, as such, may not understand the need to screen in the ED. ED staff may also be unaware of local community-based social services and lack a standardized process for connecting at-risk patients to necessary resources in the community. Though there is growing support for using existing community-based services to address social needs, coordination between clinical and community settings remains fragmented and often ineffective [19]. Cross-sector partnerships may help address social needs and are associated with improved healthcare utilization and spending [20].

This paper describes the protocol for the Building Resilience and InDependence for Geriatric Patients in the Emergency Department (BRIDGE) study, which aims to establish a simple, sustainable, and scalable ED-based process to systematically identify older patients who are at risk for malnutrition and food insecurity and link them to community-based social services to reduce risk and improve overall health and well-being.

## Methods

The screening and referral process will be developed and tested across 2 distinct phases (described in more detail below) at the University of North Carolina Hospitals Emergency Department (UNC ED), located in Chapel Hill, NC. The UNC ED provides 24-h emergency care for approximately 65,000 patients per year, including ~ 15,500 patients age 60 years and older, and serves a population that is diverse in ethnicity, socioeconomic status, and rural versus urban residence.

### Phase 1: Developing an ED-based screening and referral process

We will identify the existing screening tools, establish workflows, and conduct initial feasibility testing with UNC ED staff and patients. The primary aim is to learn how screening for malnutrition and food insecurity could best be incorporated into the existing workflow for the ED staff, including nurses, nursing assistants (NAs), and case managers (CMs). Throughout phase 1, we intend to use evidence-based strategies to guide the development and implementation [21, 22], such as the Consolidated Framework for Implementation Research (CFIR) [23] and the Plan-Do-Study-Act (PDSA) method [24], to help us quickly test, modify, and improve the screening tool and workflows. Phase 1 findings will be used to inform the ED-based screening and referral process, which will be piloted and evaluated in phase 2.

#### Identifying screeners for malnutrition and food insecurity

We will leverage existing screening tools for malnutrition and food insecurity, with consideration for the settings in which they were designed for and studied. For example, the two-item Malnutrition Screening Tool (MST) is a validated malnutrition risk screener and recommended for use among older adults in inpatient settings [25] and is already used in inpatient, acute care settings at UNC. The two-item Hunger Vital Sign (HVS) food insecurity screener is recommended for use in clinical settings [26] and recommended for use among older adults by AARP [27].

To select which screeners will be used for feasibility testing within the ED, multiple screeners will be combined into a questionnaire and tested with a small cohort of older, English-speaking patients who are 60 years of age and above and who seek care at the UNC ED. In addition to the malnutrition and food insecurity screener items, the questionnaire will assess for unmet social needs, including housing, social support, transportation, and financial strain. The questionnaire will also include items to gauge patient interest in and receptivity to receiving information about various community-based resources and assistance connecting with social services (e.g., SNAP, food pantry, congregate meal sites, home-delivered meals, transportation, and in-home meal preparation assistance). During this initial testing with patients, the questionnaire will be administered by research staff. Analyses will include descriptive statistics of patient demographics, screening questions, and additional items, such as acceptability and comprehension of screening questions, percentage of positive screens for malnutrition, food insecurity and both malnutrition and food insecurity, additional unmet social needs identified, and willingness of patients to receive assistance.

#### Engaging ED stakeholders and establishing workflows

Throughout phase 1, input will be solicited from UNC ED staff to understand their roles and associated workflows. Input will also include which ED staff roles may be best suited for this new workstream, their initial impression of the proposed screening and referral process, and any possible barriers or facilitators, including leadership buy-in. As we plan to incorporate the final screening tool and workflows into UNC’s electronic health record (EHR), Epic, we will solicit ED staff preferences related to where the screener is placed in Epic and explore the possibility of automated alerts.

Information may be collected using a variety of methods, including semi-structured informational interviews and soliciting feedback during staff meetings. Semi-structured interview guides will be developed using relevant domains and key constructs from CFIR [23], including those from the inner setting (e.g., implementation climate, compatibility, leadership engagement, tension for change) and characteristics of individuals (e.g., knowledge and beliefs about the intervention). Interviews will be conducted by the study staff, audio-recorded, and transcribed. A rapid analysis approach will be used to analyze the qualitative data [28, 29].

#### Feasibility testing with ED staff

Feasibility testing of the malnutrition and food insecurity screener and workflows will be conducted with a subgroup of older patients and appropriate UNC ED staff (e.g., NAs and CMs), potentially starting with a paper-based version of the screening tool. Rapid-cycle testing, such as PDSA [24], will be used to identify barriers and test solutions prior to full implementation. Throughout the testing, the workflows will be modified as necessary and the staff will serve as “champions” when the process is broadly implemented across the ED in phase 2.

#### Engaging community stakeholders and establishing partnerships

A critical component to the success of the study will be engaging with community stakeholders and establishing a cross-sector partnership with an Area Agency on Aging (AAA), a public or private agency appointed by the state to assist or coordinate services for older adults and persons with disabilities at the regional and local levels [30]. The AAA will accept direct referrals from the UNC ED for older patients who screen positive for malnutrition risk and/or food insecurity, follow up with patients to assess for additional resource needs, identify referral options, and provide or coordinate services.

In phase 1, we plan to identify and assess potential AAA partners and gain a better understanding of the geographic area (counties) served, availability of programs and services, service providers, program eligibility, needs assessment, capacity in the community to meet the social service needs of at-risk patients, and willingness and capabilities of the AAA to partner and share data.

During the process of establishing a partnership, we will track and document learnings, guided by CFIR [23]. This will likely include conducting semi-structured interviews to capture lessons learned and best practices about establishing a clinical-community partnership and data collection and reporting processes. In preparation for phase 2, we will work closely with the selected AAA to identify key data points, develop a plan for bidirectional data sharing, and create an efficient and reliable referral pathway.

### Phase 2: Testing the ED-based screening and referral process

In this phase, we plan to implement and evaluate the ED-based screening and referral process developed in phase 1. Older UNC ED patients who screen positive for both malnutrition risk and food insecurity will be referred to a local AAA to assess at-risk patients for additional related social needs and connect them to appropriate supports and services. Assuming approximately 10% of UNC ED older patients will screen positive (i.e., be at risk for both malnutrition and food insecurity), we plan to screen at least 2200 patients who enter the ED and enroll approximately 220 patients, using continuous screening and enrollment. Patients will not be randomized; all eligible patients will be screened, and a single-arm design will be used to evaluate the feasibility based on the proportion of patients screened and linked to services.

We will also conduct preliminary evaluations on the impact of the screening and referral process on malnutrition, general health, and other patient-reported outcomes. Details of the workflow will be determined during the formative work and testing in phase 1, though we will continue to assess the feasibility and sustainability in phase 2. Once workflows have been finalized, we will collaborate with the stakeholders at UNC’s ED and the selected AAA partner to develop protocols for each group involved (e.g., nursing, social work, and the AAA), which will serve as guides for the screening, referral, and documentation processes in phase 2.

**Fig. 1:**
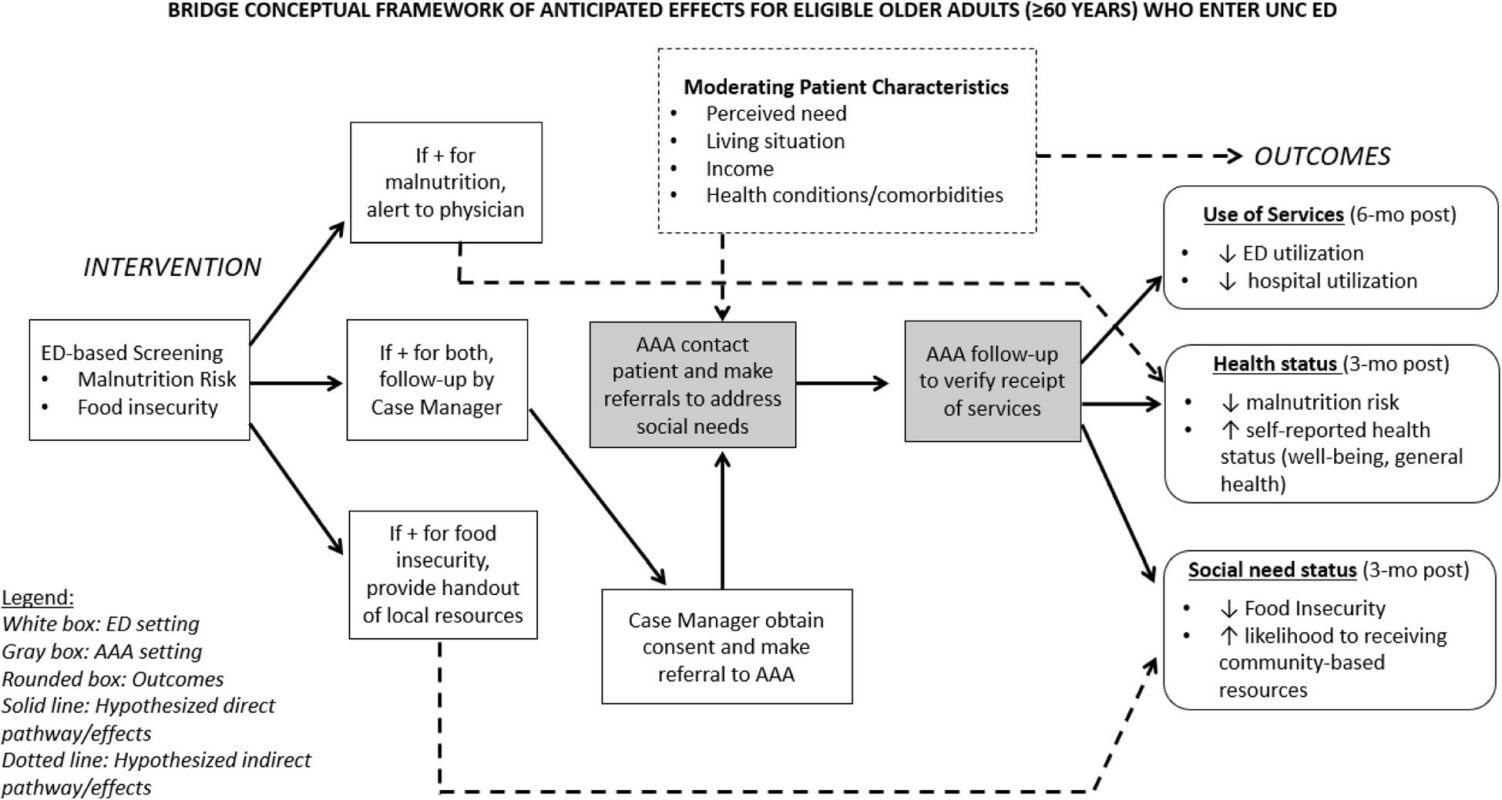
Conceptual framework of anticipated effects for eligible older adults (> 60) who are screened within the UNC ED as part of the BRIDGE study

A conceptual model of the screening and referral process is included in Fig. 1. Moving from left to right, the model shows the potential pathway (in solid arrows) for older patients who enter the ED and are screened for both malnutrition risk and food insecurity. If positive for both, the patient will be connected to a local AAA, which will follow up with the patient to assess resource needs and make referrals to community-based organizations that provide social supports and services. Funding will be available to support the care coordination work of the AAA and community-based organizations in providing services, particularly when programs have waitlists. We hypothesize that patients who are successfully connected with the needed services will have better outcomes at 3-month post, including decreased risk of malnutrition and food insecurity and decreased healthcare utilization. We anticipate that patient characteristics and clinical intervention from their primary care physician (PCP) may serve as mediators or moderators, which are depicted by dashed arrows.

#### Eligibility criteria for ED patients

Patients who are aged 60 years and older, are English speaking, and seek care in the UNC ED will be eligible to be screened. Patients with cognitive impairment may be included if a proxy is available and caregiver consent to participate is obtained. Patients will be considered ineligible if they are (1) on a psychiatric hold or (2) critically ill. Clinical judgment will be used to discern if screening is appropriate due to emotional stress or physical discomfort.

The BRIDGE study was submitted to the Institutional Review Board (IRB) as a research study with a waiver of signed informed consent for patients to receive the screening and referral process. Among patients who screen positive, verbal consent will be obtained by the ED case manager to connect patients with the AAA, which is their standard clinical practice. UNC study team members will contact the patients via phone 3-month post and will obtain verbal consent to collect follow-up outcome data at that point. To the greatest extent possible, the process developed in phase 2 will reflect existing ED screening and referral pathways.

#### Data collection and analyses

Data will be collected from multiple sources and entered by research staff into REDCap, a secure, web-based database that meets or exceeds NIH security requirements, and stored and maintained by UNC. Process outcomes will include the proportion of eligible patients for whom malnutrition and food insecurity screens are completed, the proportion of screens that are positive, the number of referrals made from the UNC ED to the AAA, the number of patients contacted by the AAA, and the number and status of referrals made by the AAA to other social service programs and providers. Healthcare utilization outcomes will be selected based on data points available in Epic and, if possible, may include a retroactive review of utilization data from 6-month pre-enrollment, as well as up to 6-month post-enrollment. Patient-reported outcomes will be collected at the time of enrollment (baseline) and 3-month post-enrollment and will likely include single items of overall health and quality of life [31]. Malnutrition and food insecurity risk will also be re-assessed with a 3-month follow-up phone call. A formal data analysis plan will be developed during phase 1, and further analysis of the data will be explored as the study progresses.

In addition, we will continue to gather stakeholder perspectives throughout the implementation to determine if the intervention is feasible and sustainable, guided by CFIR [23]. Periodic reflections with the ED and AAA staff champions or key implementers will be used to understand implementation phenomena, including unplanned adaptations and real-time barriers or facilitators [32]. Qualitative data will be collected and analyzed in a similar approach as in phase 1.

## Discussion

The purpose of the BRIDGE study is to assess the feasibility of embedding a malnutrition and food insecurity screener for older adults within an ED and referring patients who screened positive to an AAA to coordinate community-based social services. The study design, implementation, and evaluation will be guided by a prominent implementation framework (CFIR) [23], which is intended to contribute to the sustainability of the screening process and generalizability of the findings. Findings from the current study will lay the foundation for conducting larger-scale studies to assess the effect of screening for malnutrition and food insecurity and connecting patients to community-based social services. If successful, the tools and workflows developed in this study have the potential to serve as a model for future efforts to utilize EDs as a setting for bridging the gap between healthcare and social services for older adults, with the potential for improvements in health and quality of life.

The study aligns with the increasing prioritization of value-based care and geriatric-focused ED care associated with social determinants of health and addressing unmet social needs, both of which have broader implications reaching beyond the UNC ED. As in other states, North Carolina’s health system reflects this trend. For example, the UNC ED is part of the UNC Senior Alliance, a growing Next Generation Accountable Care Organization (NGACO), which provides integrated care to a subset of high-risk patients, including approximately 10% of patients ages 65 years and older seen in the UNC ED each year. Additionally, one of the largest insurers in the state, Blue Cross Blue Shield, has recently publicized its intent to have all four million members in risk-based contracts by 2024 [33]. Efforts to address malnutrition-related social needs require strong links with social services and a spectrum of care management options, ranging from intensive case management to transition calls following an ED visit, and will play a role in the success of the existing and future value-based arrangements.

### Limitations and challenges

Implementing a new screening and referral process in an ED requires a shared sense of urgency, staff buy-in, and leadership support [34]. However, we recognize that we will likely encounter competing priorities during implementation, such as other quality improvement initiatives or emergent trends in the ED, particularly those that require the time of nursing staff or care managers. Anticipating and understanding the potential barriers early on will allow us to deploy strategies that facilitate implementation [35]. For example, implementation literature cites lack of time and limited knowledge as common barriers for adoption of new procedures by frontline staff (i.e., nurses, nursing assistants, and social workers) and highlights the need for screening tools to be brief and easily integrated into standard workflows [36, 37]. As such, strategies to mitigate anticipated barriers, including identifying champions, assessing knowledge and providing training, and selecting short screeners, will be integrated into the design of the screening and referral process.

Another potential challenge is the limited availability of community-based social services and supports. Our proposed approach leverages an AAA as a resource hub where AAA staff will assess for unmet social needs and coordinate the delivery of services for the at-risk older patients identified in the ED. Currently, many senior programs in North Carolina, such as home-delivered meals, congregate meals, food pantries, grocery delivery, and transportation services, are experiencing high demand for services and have waitlists, due in part to limited funding, staff capacity, and geographical scope of their services areas. In anticipation of this, we plan to allocate short-term funding for the provision of services (e.g., covering approximately 2 months of home-delivered meals) for at-risk patients immediately following ED or hospital discharge, if needed. This approach is consistent with other post-discharge nutrition interventions that provide home-delivered meals [38, 39]. However, it is possible that some patients will return to a waitlist for services after the short-term funding is exhausted.

The demand for home-delivered meals has grown as evidence builds regarding their role in improving diet quality and reducing the use of the costliest healthcare services [15, 16], though funding for home-delivered meal programs has not kept up with the demand [14]. Through this intervention, we will learn which communities need additional capacity for the provision of senior services. The current study aims to provide evidence on the benefits of providing payments for social services for at-risk older patients.

The combination of inner-setting (e.g., lack of knowledge among front-line staff) and outer-setting (e.g., program waitlists) barriers may limit the number of patients who are screened, referred to services, and ultimately receive services. Implementation barriers in the inner setting (i.e., the ED) are likely to be the most modifiable. Thus, efforts in phase 2 will focus specifically on overcoming the implementation challenges in the ED to ensure that as many patients as possible are screened and referred to services. Data collection in phase 2 will address questions of availability of and satisfaction with services delivered by community-based organizations, as well as associations with healthcare utilization. Lessons learned from stakeholders and patients in phase 2 will inform further work and recommendations on strengthening the ties between healthcare systems and community-based organizations.

## Conclusion

The integration of care coordination approaches in healthcare and connections to social services also reflect the growing recognition of the role of social determinants of health in healthcare [40]. Amid a growing consensus on how social and environmental factors impact health, providers and health systems are developing novel techniques to identify patients’ social needs in clinical settings and link them to community-based services, a process often referred to as “social prescribing.” While there is promising data on the benefits of “social prescribing,” [41, 42] more evidence is needed to demonstrate value, which can be broadly understood as the ratio of reductions in healthcare spending to improvements in health outcomes [43]. We believe the BRIDGE study will provide important insights into how to increase access to health-related social services for vulnerable older adults.

## Data Availability

Not applicable

## Abbreviations

AAA: Area Agency on Aging
ED: Emergency department

## References

[1] Pereira GF, Bulik CM, Weaver MA, Holland WC, Platts-Mills TF (2015). Malnutrition among cognitively intact, noncritically ill older adults in the emergency department. Ann Emerg Med.

[2] Ferguson M, Capra S, Bauer J, Banks M (1998). Quality of life in patients with malnutrition. J Am Diet Assoc.

[3] Spaccavento S, Del Prete M, Craca A, Fiore P (2009). Influence of nutritional status on cognitive, functional and neuropsychiatric deficits in Alzheimer’s disease. Arch Gerontol Geriatr.

[4] Saka B, Kaya O, Ozturk GB, Erten N, Karan MA (2010). Malnutrition in the elderly and its relationship with other geriatric syndromes. Clin Nutr.

[5] Agarwal E, Ferguson M, Banks M, Batterham M, Bauer J, Capra S (2013). Malnutrition and poor food intake are associated with prolonged hospital stay, frequent readmissions, and greater in-hospital mortality: results from the Nutrition Care Day Survey 2010. Clin Nutr.

[6] Burks CE, Jones CW, Braz VA, Swor RA, Richmond NL, Hwang KS (2017). Risk factors for malnutrition among older adults in the emergency department: a multicenter study. J Am Geriatr Soc.

[7] Thomas KS, Akobundu U, Dosa D (2016). More than a meal? A randomized control trial comparing the effects of home-delivered meals programs on participants’ feelings of loneliness. J Gerontol: Series B.

[8] Agarwal E, Miller M, Yaxley A, Isenring E (2013). Malnutrition in the elderly: a narrative review. Maturitas.

[9] National Academies of Sciences E, and Medicine (NASEM) (2016). Meeting the dietary needs of older adults: exploring the impact of the physical, social, and cultural environment: workshop summary.

[10] Pooler JA, Hartline-Grafton H, DeBor M, Sudore RL, Seligman HK (2019). Food insecurity: a key social determinant of health for older adults. J Am Geriatr Soc.

[11] Berkowitz Seth A., Basu Sanjay, Meigs James B., Seligman Hilary K. (2017). Food Insecurity and Health Care Expenditures in the United States, 2011-2013. Health Services Research.

[12] Popham L (2015). Senior SNAP participation visualization: National Council on Aging.

[13] Thomas KS, Mor V (2013). Providing more home-delivered meals is one way to keep older adults with low care needs out of nursing homes. Health Aff.

[14] Sahyoun NR, Vaudin A (2014). Home-delivered meals and nutrition status among older adults. J Nutr Clin Pract.

[15] Berkowitz SA, Terranova J, Hill C, Ajayi T, Linsky T, Tishler LW (2018). Meal delivery programs reduce the use of costly health care in dually eligible Medicare and Medicaid beneficiaries. J Health Affairs.

[16] Berkowitz SA, Terranova J, Randall L, Cranston K, Waters DB, Hsu J (2019). Association between receipt of a medically tailored meal program and health care use. JAMA Intern Med.

[17] Thomas KS, Mor V (2013). The relationship between Older Americans Act Title III state expenditures and prevalence of low-care nursing home residents. Health Serv Res.

[18] Pines JM, Hollander JE (2008). Emergency department crowding is associated with poor care for patients with severe pain. Ann Emerg Med.

[19] Sahyoun NRAU, Sharkey JR, Netterville L (2010). Recently hospital-discharged older adults are vulnerable and may be underserved by the Older Americans Act Nutrition Program. J Nutr Elder.

[20] Brewster AL, Kunkel S, Stranker J, Curry LA (2018). Cross-sectoral partnerships by area agencies on aging: associations with health care use and spending. Health Aff.

[21] Tabak RG, Khoong EC, Chambers DA, Brownson RC (2012). Bridging research and practice: models for dissemination and implementation research. Am J Prev Med.

[22] Center for Research in Implementation Science and Prevention (CRISP) 2018. Available from: http://www.dissemination-implementation.org/index.aspx. Accessed 20 Aug 2019.

[23] Damschroder LJ, Aron DC, Keith RE, Kirsh SR, Alexander JA, Lowery JC (2009). Fostering implementation of health services research findings into practice: a consolidated framework for advancing implementation science. J Implement Sci.

[24] Taylor MJ, McNicholas C, Nicolay C, Darzi A, Bell D, Reed JE (2014). Systematic review of the application of the plan–do–study–act method to improve quality in healthcare. BMJ Qual Saf.

[25] Young AM, Kidston S, Banks MD, Mudge AM, Isenring EA (2013). Malnutrition screening tools: comparison against two validated nutrition assessment methods in older medical inpatients. Nutrition.

[26] Makelarski JA, Abramsohn E, Benjamin JH, Du S, Lindau ST (2017). Diagnostic accuracy of two food insecurity screeners recommended for use in health care settings. Am J Public Health.

[27] Pooler J, Levin M, Hoffman V, Karva F, Lewin-Zwerdling A (2016). Implementing food security screening and referral for older patients in primary care: a resource guide and toolkit.

[28] Keith RE, Crosson JC, O’Malley AS, Cromp D, Taylor EF (2017). Using the Consolidated Framework for Implementation Research (CFIR) to produce actionable findings: a rapid-cycle evaluation approach to improving implementation. J Implement Sci.

[29] Ash JS, Sittig DF, CK MM, Guappone K, Dykstra R, Carpenter J, editors. A rapid assessment process for clinical informatics interventions, AMIA Annual Symposium Proceedings. Bethesda: American Medical Informatics Association; 2008. PMC2656056 18999075

[30] Locator E. Area Agencies on Aging. Available from: https://eldercare.acl.gov/Public/About/Aging_Network/AAA.aspx. [cited 2019 June 27 ].

[31] Cella D, Riley W, Stone A, Rothrock N, Reeve B, Yount S (2010). The Patient-Reported Outcomes Measurement Information System (PROMIS) developed and tested its first wave of adult self-reported health outcome item banks: 2005–2008. J Clin Epidemiol.

[32] Finley EP, Huynh AK, Farmer MM, Bean-Mayberry B, Moin T, Oishi SM (2018). Periodic reflections: a method of guided discussions for documenting implementation phenomena. BMC Med Res Methodol.

[33] Sharp JP, Patrick C, Rahul R. Engineering a Rapid Shift to Value-Based Payment in North Carolina: Goals and Challenges for a Commercial ACO Program. N Engl J Med. 2019. Available from: https://catalyst.nejm.org/blue-premier-nc-value-based-payment/.

[34] Kotter JP (1996). Why transformation efforts fail. J Prod Innov Manag.

[35] Powell BJ, Waltz TJ, Chinman MJ, Damschroder LJ, Smith JL, Matthieu MM (2015). A refined compilation of implementation strategies: results from the Expert Recommendations for Implementing Change (ERIC) project. Implement Sci.

[36] Louwers ECFM, Korfage IJ, Affourtit MJ, De Koning HJ, Moll HA (2012). Facilitators and barriers to screening for child abuse in the emergency department. BMC Pediatr.

[37] Tiyyagura G, Schaeffer P, Gawel M, Leventhal JM, Auerbach M, Asnes AG (2019). A qualitative study examining stakeholder perspectives of a local child abuse program in community emergency departments. Acad Pediatr.

[38] Hummel SL, Karmally W, Gillespie BW, Helmke S, Teruya S, Wells J (2018). Home-delivered meals postdischarge from heart failure hospitalization. Circ Heart Fail.

[39] Buys DR, Campbell AD, Godfryd A, Flood K, Kitchin E, Kilgore ML (2017). Meals enhancing nutrition after discharge: findings from a pilot randomized controlled trial. J Acad Nutr Diet.

[40] Alderwick HAJ, Gottlieb LM, Fichtenberg CM, Adler NE (2018). Social prescribing in the U.S. and England: emerging interventions to address patients’ social needs. Am J Prev Med.

[41] Gottlieb LM, Wing H, Adler NE (2017). A systematic review of interventions on patients’ social and economic needs. J Am J Prev Med.

[42] Bickerdike L, Booth A, Wilson PM, Farley K, Wright K (2017). Social prescribing: less rhetoric and more reality. A systematic review of the evidence. BMJ Open.

[43] Porter ME (2010). What is value in health care?. N Engl J Med.

